# Recurrent Diplopia in a Non-Smoking Young Adult with Clubbing: A Case to Remember

**DOI:** 10.51894/001c.144421

**Published:** 2025-09-30

**Authors:** Syeda Juvaria Arshi

**Affiliations:** 1 Department of Internal Medicine Trinity Health Oakland

## 64

### INTRODUCTION

Stroke in young adults, though rare, can have devastating consequences. Cryptogenic strokes, often associated with structural cardiac abnormalities like Patent Foramen Ovale (PFO), pose a significant diagnostic challenge. PFO, present in 25% of the population, can allow paradoxical embolism under certain conditions, causing ischemic events. This case examines whether early detection and management of PFO in a 29-year-old male with acute-onset binocular diplopia and cryptogenic stroke could have prevented his ischemic event.

### METHODS

The patient presented with acute painless binocular diplopia, a history of a transient similar episode three years earlier, and grade 3 nail clubbing. Imaging identified an acute, non-hemorrhagic stroke involving the right thalamus, subthalamic nucleus, and red nucleus. Echocardiography confirmed a Grade III PFO with bidirectional shunting and lipomatous hypertrophy of the interatrial septum. Elevated lipoprotein (a) and a positive THC toxicology screen were noted. The patient was managed with antiplatelet therapy, statins, and discharged with plans for PFO closure and hypercoagulable workup.

### DISCUSSION

This case highlights the missed opportunity for earlier PFO detection during the patient’s previous transient diplopia episode. The presence of grade 3 clubbing, a potential cardiovascular clue, was overlooked. PFO closure has shown efficacy in reducing recurrent strokes in cryptogenic stroke patients with high-risk PFO characteristics. Factors like elevated lipoprotein (a) further increased the risk of paradoxical embolism. The case also underscores the challenges of atypical presentations, lack of standardized screening, and reliance on advanced diagnostics over physical examination.

### CONCLUSION

This case emphasizes the importance of early recognition of subtle neurological symptoms, comprehensive evaluation of at-risk individuals, and prompt management of PFO to prevent strokes in young adults. Reinforcing physical examination skills, raising public awareness, and establishing screening guidelines for high-risk populations are vital for improving outcomes in cryptogenic strokes. Further research is needed to refine risk stratification and cost-effective screening approaches.

